# Pudendal nerve decompression in perineology : a case series

**DOI:** 10.1186/1471-2482-4-15

**Published:** 2004-10-30

**Authors:** Jacques Beco, Daniela Climov, Michèle Bex

**Affiliations:** 1 Gynaecology, CHU Sart-Tilman, University of Liège, B-4000 Liège, Belgium; 2 Perineology, CHC-Clinique Sainte-Elisabeth, 17 rue du Naimeux, B-4802 Heusy, Belgium; 3 Research, Institut d'Enseignement Supérieur Parnasse-Deux Alice, Avenue Mounier 84, B-1200 Brussels, Belgium; 4 Physiotherapy, CHR La Citadelle, Boulevard du 12^ème ^de Ligne, B-4000 Liège, Belgium

## Abstract

**Background:**

Perineodynia (vulvodynia, perineal pain, proctalgia), anal and urinary incontinence are the main symptoms of the pudendal canal syndrome (PCS) or entrapment of the pudendal nerve. The first aim of this study was to evaluate the effect of bilateral pudendal nerve decompression (PND) on the symptoms of the PCS, on three clinical signs (abnormal sensibility, painful Alcock's canal, painful "skin rolling test") and on two neurophysiological tests: electromyography (EMG) and pudendal nerve terminal motor latencies (PNTML). The second aim was to study the clinical value of the aforementioned clinical signs in the diagnosis of PCS.

**Methods:**

In this retrospective analysis, the studied sample comprised 74 female patients who underwent a bilateral PND between 1995 and 2002. To accomplish the first aim, the patients sample was compared before and at least one year after surgery by means of descriptive statistics and hypothesis testing. The second aim was achieved by means of a statistical comparison between the patient's group before the operation and a control group of 82 women without any of the following signs: prolapse, anal incontinence, perineodynia, dyschesia and history of pelvi-perineal surgery.

**Results:**

When bilateral PND was the only procedure done to treat the symptoms, the cure rates of perineodynia, anal incontinence and urinary incontinence were 8/14, 4/5 and 3/5, respectively. The frequency of the three clinical signs was significantly reduced. There was a significant reduction of anal and perineal PNTML and a significant increase of anal richness on EMG. The Odd Ratio of the three clinical signs in the diagnosis of PCS was 16,97 (95% CI = 4,68 – 61,51).

**Conclusion:**

This study suggests that bilateral PND can treat perineodynia, anal and urinary incontinence. The three clinical signs of PCS seem to be efficient to suspect this diagnosis. There is a need for further studies to confirm these preliminary results.

## Background

The objective of perineology is to treat each defect of the perineum with the right procedure [1-3]. Pudendal nerve decompression (PND) is theoretically a basic procedure in perineology thanks to its ability to treat the defect "pudendal neuropathy".

Before going into details of this procedure, it is necessary to remember the anatomy of the pudendal nerve. This anatomy is still controversial.

While summarizing the data of the literature and the results of our dissections, the likeliest anatomy of the pudendal nerve presents itself as follows. The pudendal nerve is a mixed nerve carrying motor and sensory fibers. Its fibers are derived from the sacral roots S2, S3 and S4 [4,5]. Once the roots traverse the sacral foramen, they divide into autonomic branches forming the pelvic plexus (parasympathetic supply of the pelvic organs) and somatic branches merging to form the pudendal nerve travelling under the piriformis muscle. Near its formation point, it gives a levator branch running on the inner (upper) surface of the levator plate and providing the innervation of this muscle [4]. For Barber et al [6], this levator nerve originates directly from the S3, S4 or S5 roots. Some somatic fibers coming from S2 and S3 run close to the pelvic plexus to innervate the levator ani and the urethral sphincter [4]. Caudally, the pudendal nerve enters a small space ("clamp") between the sacro-spinal and sacro-tuberous ligaments very near the ischial spine. Just inferior to the ischial spine, the nerve gives its first branch, the dorsal nerve of the penis [4] or the clitoridal nerve. These nerves are separated from the main trunk by the pudendal vein and artery. Then, it enters the Alcock's canal formed by a division of the obturator muscle aponeurosis. In the canal the nerve crosses the sharp edge of the sacro-tuberous ligament (falciform process) [7,8]. Caudally, at the level of the anus, the nerve gives medially the inferior rectal nerves (usually two branches) which innervate the anal sphincter (and probably the pubo-rectalis) and the skin of the posterior perineum and anterolaterally the transversus perinei branch (for this muscle, for the ischiocavernosus muscle and maybe for the urethral sphincter) [4]. The remaining part of the nerve is usually called the perineal nerve. This nerve gives a bulbocavernosus branch and finally divides into a sphincteric branch (innervation of the urethra) and a branch which innervates the skin of the anterior perineum [9].

The pudendal canal syndrome (PCS) and its surgical treatment have been described by Shafik in 1991 [5]. This syndrome is induced by the compression or the stretching of the pudendal nerve in the Alcock's canal. The complete syndrome presents with anal incontinence, pain, hypo or hyperesthesia and urinary incontinence (and impotence in males). Some important studies were done earlier by Amarenco [10] and Robert [7] but these authors focused mainly on pain which is only a part of the syndrome.

The cause of the PCS is not always clear but it is often possible to find a compression (biking, long time sitting, haematoma...) or a stretching (descending perineum, surgery, delivery....) of the pudendal nerve in the Alcock's canal [10-19] in the history of the patient. A change in the shape or orientation of the ischial spine induced by some athletic activities during the youth could also explain some cases [20].

The clinical signs and investigation results proposed by Shafik [5] to confirm the diagnosis of PCS before surgery are: tenderness over the pudendal canal in the ischio-rectal fossa, diminished perineal sensation, weak or absent anal reflex, reduced EMG activity of the external anal sphincter and increased PNTML. The surgical procedure described by this author (trans-perineal approach) consists in opening the Alcock's canal to give the pudendal nerve a sufficient length to be unstretched and/or to suppress compression.

The trans-gluteal approach proposed by Robert to treat pudendal neuralgia aimed to open also the "clamp" between the sacro-spinal and the sacro-tuberous ligament by cutting one or two of them [8].

Since Shafik's study in 1991, some questions about the effect of PND on the PCS remained open. No peer reviewed publications confirming the efficacy of this surgery on anal incontinence or on urinary incontinence could be found. Even the existence of a genuine PCS has never been validated.

The aim of this study was to answer the following questions:

Is there any effect of the bilateral PND according to Shafik on:

- three main symptoms of PCS ?

           - perineodynia (vulvodynia, perineal pain, proctalgia) [21]

           - anal incontinence

           - urinary incontinence

- three clinical signs of PCS ?

           - abnormal sensibility

           - painful Alcock's canal

           - painful "skin rolling test" [22]

- two neurophysiological tests ?

           - electromyography (EMG) of the anal sphincter and of the bulbocavernosus muscles.

           - pudendal nerve terminal motor latencies (PNTML) of the anal and perineal branches.

What is the clinical value of the aforementioned three clinical signs in the diagnosis of PCS ?

## Methods

### Studied sample

A retrospective analysis to study the effects of a bilateral PND. The studied sample comprised 74 female patients who underwent a bilateral PND between 1995 and 2002 done by the same surgeon. The average age was 56.1 years (range: 28 – 77). All these patients underwent a complete history and clinical examination following the three perineal axis (gynaecological, urological and colo-proctological) according to the concept of perineology [1-3].

**Figure 1:**
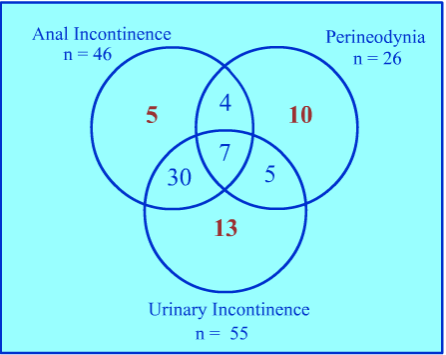
Frequency of the 3 main symptoms of the pudendal canal syndrome (perineodynia, anal incontinence, urinary incontinence) before surgery.

The frequency of the 3 main symptoms of the PCS (anal incontinence, perineodynia, urinary incontinence) in the 74 patients before surgery is presented in Figure 1.

Associated surgical procedures were performed at the same time as the PND to treat all the defects revealed by the clinical examination, and are presented in Table 1.

**Table 1:** Procedures associated with the bilateral pudendal nerve decompression

**Associated procedures**	**Operated (n = 74)**	**Reviewed after one year or more (n = 56)**
None	17	10
MVT according to Mouchel [48-50]	46	38
Correction of rectocele	49	42
Correction of cystocele	20	17
Vaginal hysterectomy	16	13
Levatorplasty according to Shafik [26]	14	13
Urethral meatotomy	4	3
Prepubien section [45]	2	1
Anal sphincteroplasty	2	2
Urethrolysis	1	1

### Diagnostic tools for PCS

The following variables were used:

#### Three main symptoms of the PCS

##### Perineodynia

For perineodynia, four situations were encountered: no pain, proctalgia, unilateral pain, bilateral pain. The effect of surgery was estimated by the patient using one of the following proposals: cured, improved, unchanged or worsened.

##### Anal incontinence

For anal incontinence, a four levels ordinal scale was used: no incontinence, gas incontinence, liquid incontinence, solid incontinence. "Cured" was defined as "no incontinence". The patient was considered "improved" if there was a change of at least one level in the scale going from "solid" to "gas" incontinence. The patient was defined as "worsened" if there was a change of at least one level in the opposite direction.

##### Urinary incontinence

For urinary incontinence, a four levels ordinal scale was used: no incontinence, mild incontinence, moderate incontinence and severe incontinence. The two types of urinary incontinence, stress and urge incontinence, were evaluated separately even if both were present in the same patient (mixed incontinence). "Cured" was defined as "no incontinence". The patient was considered "improved" if there was a change of at least one level in the scale going from "severe" to "mild" incontinence. If the change observed was in the opposite direction the patient was considered "worsened".

#### Three clinical signs of the PCS (the examinations were done in gynaecological position)

##### Abnormal anal or vulvar sensibility

Sensibility was tested with a needle comparing the left and the right sides of the vulva and of the skin 2 cm lateral to the anus. The interpretations of the results were done using a four levels ordinal scale: 0 = total anaesthesia, 1 = reduced sensibility, 2 = normal sensibility, 3 = hypersensibility. 0, 1 and 3 were considered as "abnormal sensibility".

##### Painful Alcock's canal on rectal examination

The pain induced by the palpation of the pudendal canal by rectal examination was evaluated using a seven levels ordinal scale : 0 = no pain, 1 = mild pain, 2 = mild pain with Tinel sign (irradiation of the pain), 3 = moderate pain, 4 = moderate pain with Tinel sign, 5 = severe pain, 6 = severe pain with Tinel sign. The Alcock's canal was considered "painful" if the pain was 4 or more.

##### Painful "skin rolling test"

**Figure 2:**
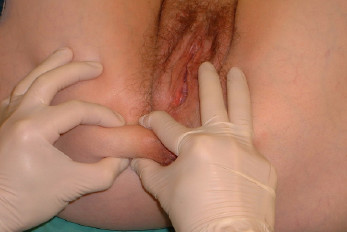
Skin rolling test : the skin of the perineum is pinched just beneath the level of the anus and then rolled to the front searching for a sharp pain at one level. This sign is well known in the diagnosis of neuralgia.

Beginning from 5 cm behind the level of anus the skin was pinched and then rolled to the front until the skin fold was at the level of the clitoris. The skin rolling test was considered "painful" if it induced a severe pain at least at one level (Figure 2).

#### Two neurophysiological tests

##### Concentric needle EMG

Concentric needle EMG was done at rest and during voluntary contraction on both sides of the external anal sphincter and on each bulbocavernosus muscle. The richness of the EMG was grossly evaluated using a six levels scale: 1 = simple, 2 = poor, 3 = intermediate poor, 4 = intermediate, 5 = intermediate rich and 6 = rich.

##### Anal and perineal PNTML

Anal and perineal PNTML were measured before and after the operation using the St Marks electrode to stimulate the pudendal nerve by the rectal route just under the ischial spine. For anal PNTML the electrical potentials induced in the striated anal sphincter were collected using the ring of this electrode. For the perineal PNTML the method described by Kiff [23] and Snooks [24] was modified according to Amarenco [25]. The electrical potentials were collected with a concentric needle in the two bulbocavernosus muscles. In our laboratory the normal values were: less than 2.5 msec for the anal PNTML and less than 5 msec for the perineal PNTML. EMG and PNTML were done by the same physician before and at least one year after the operation.

### Minimal criteria for surgery

At least one of the 3 following symptoms resistant to conservative treatments (physiotherapy, drugs, infiltrations, modification of diet or behaviour):

a. Anal incontinence

b. Perineodynia

c. Urinary incontinence

Associated with at least two of the five following criteria:

a. increased anal or perineal PNTML

b. pathological EMG of the anal sphincter or bulbocavernosus muscles (neurogenic trace, reduced activity: richness "poor" or "simple").

c. painful Alcock's canal on rectal examination (at least on one side)

d. abnormal perineal sensibility (at least at one level)

e. painful "skin rolling test" (at least on one side).

### Surgical procedure

Surgical procedure as described by Shafik in 1991 [5]. The operations were done under spinal or general anaesthesia. The patients were installed in the gynaecological position.

**Figure 3:**
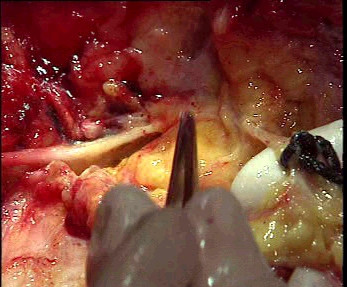
Left Alcock's canal (showed by the tip of the forceps) viewed from the mid side on a female cadaver: on the left the pudendal nerve, on the right the inferior rectal nerve on the finger.

**Figure 4:**
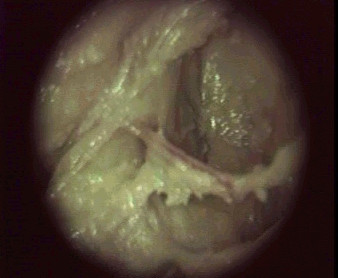
Alcock's canal viewed from below like in the operating room (right side of a female cadaver): inferior rectal nerve (horizontal) showing the entrance of the canal.

The different steps of the procedure were:

- Vertical incision of the skin between the anus and the ischial tuberosity.

- Opening of the ischio-rectal fossa with scissors.

- The inferior rectal nerve is hooked under the finger and followed to the entrance of the Alcock's canal (Figures 3 and 4).

- Opening of this canal (without opening the clamp between the sacro-spinal and sacro-tuberous ligament).

- Control of the haemostasis.

- Self draining closing of the skin with nylon.

### Evaluation of PND

The efficacy on the symptoms, on the clinical signs and on the neurophysiological tests was evaluated during a follow up consultation one year or more after the surgical procedure because the nerve healing can be very slow [5].

### Statistical methods

Firstly, the efficiency of the PND on the symptoms and clinical signs was studied by means of descriptive statistics. Tests of hypothesis were done to compare the mean values of the neurophysiological tests before versus after PND.

Secondly, the diagnostic value of the clinical signs was evaluated in a "case control" setting. A subject belongs to the "controls group" when PCS is considered to be absent, namely if the patient does not present any of the following symptoms, signs or risk factors for PCS: perineodynia, anal incontinence, prolapse, previous surgery in the area, dyschesia. The clinical signs are not used to decide if a subject belongs to the controls or cases group. The statistical comparison was done between the patient's group before ("cases group") and after the operation, and the "controls group" of 82 women (average age: 48.8 years, extremes 27–76).

Statistical analysis of differences was performed using chi-square testing for categorical variables and t-tests for continuous variables.

## Results

### Effects of surgery

#### Effect on the symptoms of the PCS

The effect of PND on the symptoms of PCS is presented in Table 2.

**Table 2:** Effect of PND on the 3 main symptoms of the PCS

**Parameters**	**Perineodynia (pain)**	**Perineodynia (pain)**	**Anal incontinence**	**Anal incontinence**	**Stress urinary incontinence**	**Stress urinary incontinence**	**Urge incontinence**	**Urge ** **incontinence**
	All	Without: levat	All	Without: sphincteroplasty, levat, recto	All	Without: levat, mvt, cysto, prepubien, meato, urethrolysis	All	Without: levat, mvt, cysto, prepubien, meato, urethrolysis

Number of cases studied	74	59	74	22	74	22	74	22
Number of pathological results	26	22	46	9	47	4	33	4
Follow up less than 1 year or lost	8	8	10	4	10	3	6	0
Follow up 1 year or more	18	14	36	5	37	1	27	4
Mean follow up in months (range)	22,2 (12–48)	24,5 (12–48)	26,4(12–70)	17,2 (12–26)	32 (12–96)	12	26,7(12–72)	18,5 (12–26)
Cured (%)	11 (61,1%)	8 (57,1%)	23 (63,9%)	4 (80%)	26 (70%)	0 (0%)	17 (62,9%)	3 (75%)
Improved (%)	3 (16,6%)	2 (14,3 %)	7 (19,4%)	1 (20%)	7 (18,9%)	1 (100 %)	6 (22,2%)	0 (0%)
No change (%)	4 (22,2%)	4 (28,6%)	4 (11,1%)	0 (0%)	4 (10,8 %)	0 (0 %)	3 (11,1%)	0 (0%)
Worse (%)	0 (0 %)	0 (0 %)	2 (5,5%)	0 (0%)	0 (0%)	0 (0 %)	1 (3,7%)	1 (25%)

levat = levatorplasty, recto = cure of rectocele, cysto = cure of cystocele, prepubien = prepubien section, meato = meatotomy.

In order to treat completely the patient, PND was frequently associated with other procedures which might have an effect on the symptom studied. Therefore, the results for each symptom were presented in two columns: the first corresponds to the entire sample ("all") and the second to the small group of patients in which the symptom was treated by PND only ("without").

#### Effect on perineodynia

On the 26 patients with pain before the operation, 18 were reviewed 12 months or more after the operation. The pain had disappeared in 11 and was reduced or had another origin (painful puborectalis) in 3. The cure rate with a mean follow-up of 22,2 months was 61,1 % (77,7% cured or improved).

As none of the surgical associated procedures used in this study were known to improve or cure perineodynia, the only procedure removed was levatorplasty. Theoretically this operation can reduce the stretching on the pudendal nerves by reducing the sagging of the levator plate. The results were similar in the group without levatorplasty.

#### Effect on anal incontinence

On the 46 patients with anal incontinence before the operation, 36 were reviewed 12 months or more after the operation. 23 of them were cured, 7 improved, 2 were worse and 4 reported no change. The cure rate with a mean follow-up of 26.4 months was 63.9 % (83,3% cured or improved).

The results according to the severity of incontinence are presented in Table 3.

**Table 3:** Effect of PND on anal incontinence according to the incontinence level.

	Cured	Improved	Unchanged	Worsened
Solid (n = 5)	3	2	0	0
Liquid (n = 20)	12	5	3	0
Gas (n = 11)	8	0	1	2

To study the real impact of PND on anal incontinence, we removed the following cases in which PND was associated with anal sphincteroplasty, levatorplasty or cure of rectocele by fascia and perineal body restoration:

- 2 patients had anal sphincteroplasty together with the PND: one was cured and the other improved.

- Levatorplasty according to Shafik [26] was used in 8 patients who had a severe levator plate sagging. This procedure could have a "post-anal repair effect" [27] and therefore improve anal incontinence.

- 30 cases had a cure of rectocele by fascia and perineal body restoration (Ayabaca and coll. [28] found an improvement of anal incontinence in 25 of their 34 patients. Nevertheless, none of them gained full continence post-operatively).

In the small group of patients with PND only, 5 were reviewed one year or more after the operation: 4 were cured (3 incontinences for liquid and 1 for gas) and 1 improved (liquid incontinence).

Anal ultrasound was done before the operation in 13 of the 36 patients reviewed. Only 4 were normal, 3 showed a rupture of the internal and external sphincters, and 6 presented a disruption of the external sphincter alone.

In the 7 patients who had a rupture of the anal sphincter (5 external only, 2 internal and external) without anal sphincteroplasty and a follow up of more than 12 months (mean 18.5 months), 4 were cured and 3 were a failure.

5 patients who were still incontinent after 1 year follow up (2 incontinences for flatus and 3 for liquid) became continent 2 years after the operation.

#### Effect on urinary incontinence

Five patients presenting urinary incontinence (4 urge and 1 stress incontinence) had PND without any other procedure around the urethra. The mean follow up was 18,5 months for the 4 patients with urge incontinence. In this small group, 3 patients were cured and one was a failure. The patient with stress urinary incontinence was improved (the number of pads used per day was reduced from 9 to 4) one year after surgery.

#### Effect on the clinical signs

The effect of PND on the three clinical signs is described in Table 4.

**Table 4:** Effect of PND on the three clinical signs

**Parameters**	**Abnormal sensibility (at least at one level)**	**Abnormal sensibility (at least at one level)**	**Painful Alcock's canal (at least on one side)**	**Painful Alcock's canal (at least on one side)**	**Painful skin rolling test (at least on one side)**	**Painful skin rolling test (at least on one side)**
	All	Without: levat	All	Without: levat	All	Without: levat

Number of tests before surgery	42	27	46	32	39	26
Follow up less than 1 year or lost	11	9	19	16	22	16
Follow up 1 year or more	31	18	27	16	17	10
Normal test before (reviewed 1 year or more after)	15	8	9	6	8	2
Abnormal test before (reviewed 1 year or more after)	16	10	18	10	9	8
Mean follow up (range)	27,7 (12–68)	32,2 (12–68)	28,1 (12–68)	32,8 (12–68)	28,7 (12–60)	33,7 (12–60)
Normal before => Normal after	14	8	9	6	7	2
Normal before => Abnormal after (%)	1	0	0	0	1	0
Abnormal before => Normal after (%)	11 (68%)	6 (60%)	11 (61%)	7 (70%)	6 (66,6%)	5 (62,5)
Abnormal before => Abnormal after	5	4	7	3	3	3

Because levatorplasty can theoretically reduce the stretching of the pudendal nerve, the evaluation of the effect on the clinical signs has been done for the entire sample ("all") and for the same group without the patients who had a levatorplasty ("without: levat").

The cure rate of the 3 clinical signs was between 60 and 70 % depending of the sign and of the type of sample studied.

#### Effect on EMG and PNTML

38 patients underwent a complete EMG evaluation before and after surgery. A relevant comparison was possible in 35 patients. 3 patients were excluded because one of the two EMG explorations was insufficient for technical reasons. The average follow up was 16,9 months (range: 12 – 35,4 months).

Left and right values for one parameter correspond to two different nerves. Therefore, these values were considered independent (maximum number of analysed cases: 35 × 2 = 70).

The effect of PND on EMG and PNTML is presented in Table 5.

**Table 5:** Effect of PND on EMG and PNTML

	**Anal Richness (range 1 to 6)**	**BC Richness (range 1 to 6)**	**Anal PNTML (msec)**	**Perineal PNTML (msec)**
All subjects	74	74	74	74
Follow up less than 12 months or lost	39	39	39	39
Analysed cases(left and right)	70	61	70	51
Mean Before	2,70	2,23	3,38	5,63
Mean After	3,11	2,44	2,63	5,21
t-test p-value(one-tail)	0,00007	0,06989	0,00004	0,00816

Left and right values for each level (anal and perineal) were included in the same group. P-values at the bottom line correspond to one-tailed significance tests of the mean differences "before" versus "after".

The "Anal Richness" on EMG after surgery was significantly higher than before. The mean "Bulbocavernosus Richness" after surgery was slightly higher than before but this difference was not significant. Both anal and perineal PNTML after PND were significantly reduced compared to values before.

**Figure 5:**
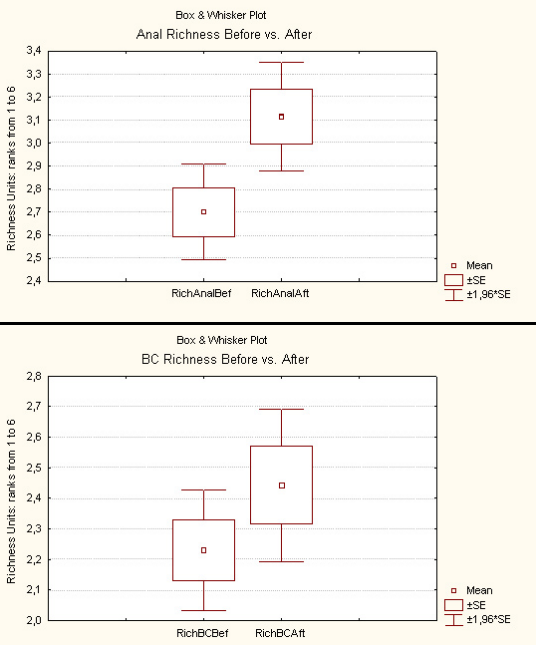
Effect of PND on anal and bulbocavernosus (BC) richness on EMG. The box is defined by the sample mean plus or minus one standard error of the sample mean. The probability to obtain a value in the box is 67 %. The whiskers represent the 95% confidence intervals of the population means.

**Figure 6:**
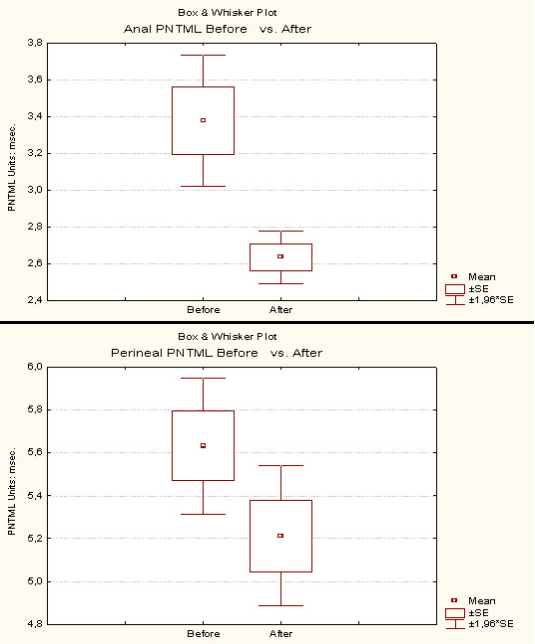
Effect of PND on anal and perineal PNTML. The box-plots definitions are the same as in Figure 5.

The box-plots of the 4 studied parameters are presented in Figures 5 and 6.

### Evaluation of the clinical signs

We present here the results concerning the evaluation of the three clinical tests: abnormal sensibility, painful Alcock's canal and painful "skin rollling test" as diagnostic tests for PCS.

The statistical analysis is based on the following contingency tables presented in Tables 6 to 9.

**Table 6:** "Abnormal sensibility" in the diagnosis of pudendal canal syndrome

	**Cases**		**Controls**
**Abnormal sensibility**	Before Surgery	After Surgery	

Abnormal	24	7	19
Normal	18	36	63
Total	42	43	82
Chi-square versus Controls	12,691	0,449	
P-value	< 0,001	0,503	

At the bottom lines, p-values and chi-square test statistics correspond to the homogeneity tests comparing the "Cases Before Surgery" to "Controls". The results obtained for the homogeneity test comparing "Cases After Surgery" versus "Controls" are also reported in the tables, although this does not contribute to the sensibility/specificity analysis.

**Table 7:** "Painful Alcock's canal" in the diagnosis of pudendal canal syndrome

	**Cases**		**Controls**
**Painful Alcock's canal**	Before Surgery	After Surgery	

Abnormal	32	10	24
Normal	14	30	58
Total	46	40	82
Chi-square versus Controls	17,842	0,0776	
P-value	< 0,001	0,781	

**Table 8:** "Painful skin rolling test" in the diagnosis of pudendal canal syndrome.

	**Cases**		**Controls**
**Painful skin rolling test**	Before Surgery	After Surgery	

Abnormal	21	6	13
Normal	17	28	69
Total	38	34	82
Chi-square versus Controls	17,968	0,001	
P-value	< 0,001	0,97	

**Table 9:** The three clinical signs in the diagnosis of pudendal canal syndrome

	**Cases**		**Controls**
**The three clinical signs**	Before Surgery	After Surgery	

Abnormal – All positive	13	3	6
Abnormal – Two positive	8	3	9
Abnormal – One positive	5	3	20
Normal – All negative	6	24	47
Total	32	33	82
Chi-square versus Controls	26,528	3,834	
P-value	< 0,001	0,280	

The proportions of observations in the "Cases Before Surgery" and "Controls" columns of the contingency table vary significantly from row to row (p-values <0,001), whereas no significant difference is observed between the proportions of observations between "Cases After Surgery" and "Controls" (p-values >0,05).

Estimated sensibility, specificity, predictive values (positive and negative) and odd ratio (estimated values and 95% confidence intervals) corresponding to each of the three clinical tests and combinations of all of them are presented in Tables 10 and 11, respectively. The predictive values were calculated for a PCS prevalence of 20 %. All the indicators in Tables 10 and 11 are estimated from data in Tables 6 to 9, columns "Cases Before Surgery" and "Controls".

**Table 10:** Evaluation of each of the three clinical signs of pudendal canal syndrome

**Tests**	**SE**	**SP**	**PPV**	**NPV**	**OR**	**95%IC(OR)**	**Nb of Cases Before**	**Nb of Controls**
Abnormal sensibility	0,57	0,77	0,38	0,88	4,42	1,99 – 9,82	42	82
Painful Alcock's canal	0,70	0,71	0,37	0,90	5,52	2,51 – 12,15	46	82
Painful skin rolling test	0,55	0,84	0,47	0,89	6,56	2,74 – 15,68	38	82

SE = sensibility, SP = specificity, PPV = positive predictive value, NPV = negative predictive value, OR = odd ratio, 95%IC (OR) = 95 % confidence interval of the odd ratio. PPV and NPV for a prevalence of 20 %.

**Table 11:** Evaluation of different combinations of the three clinical signs of pudendal canal syndrome

**Test: All Three Clinical Signs**	**SE**	**SP**	**PPV**	**NPV**	**OR**	**95%IC(OR)**	**Nb of Cases Before**	**Nb of Controls**
All positive vs. All negative	0,68	0,89	0,60	0,92	16,97	4,68 – 61,51	19	53
At least 2 positive vs. At least 2 negative	0,66	0,82	0,47	0,90	8,53	3,40 – 21,39	32	82
At least 1 positive vs. All negative	0,81	0,57	0,32	0,92	5,82	2,16 – 15,66	32	82

SE = sensibility, SP = specificity, PPV = positive predictive value, NPV = negative predictive value, OR = odd ratio, 95%IC (OR) = 95 % confidence interval of the odd ratio. PPV and NPV for a prevalence of 20 %.

The most sensible test is the "Painful Alcock's canal" and the most specific is the "Skin rolling test".

Using the three signs altogether, the most sensible combination is "At least 1 positive versus All negative" and the most specific combination is the "All positive versus All negative".

### Side effects

During one operation a heavy bleeding coming from the pudendal artery just near the pudendal nerve was very difficult to treat (selective ligature). This patient had a blood transfusion but no long term side effect. Since the operation, one patient has presented sometimes a short lasting clitoridal pain. This patient had also an increase in her anal incontinence (gas incontinence became a liquid incontinence). Three patients had wound healing problems which resolved with simple disinfection.

### Prevalence

In the literature there is no data available about the prevalence of the PCS. Therefore, it seems to be a rare event. In this study, we evaluated the prevalence of the PCS in an outpatient perineology clinic. By using three different methods during the last 24 months (percentage of pudendal nerve decompressions in the treatment of prolapse or incontinence : 13/78; percentage of anal incontinence and/or perineodynia in our outpatient consultation : 78/316 ; percentage of positive skin rolling tests : 9/55) the estimated prevalence should be around 20%.

## Discussion

### Effects of surgery

Before discussing the results of surgery, the first important issue is about the interest of a bilateral decompression. The benefit – risk ratio must be studied. The results of Shafik were obtained after bilateral decompression [5,29,30]. For urinary and anal incontinence, it seems logical to treat both nerves because the sphincters have a bilateral innervation and if one nerve is suffering maybe there is a problem on the other. The EMG exploration is not very sensitive and doesn't study the sensory pathway, which could be very important for continence. For the same reason, bilateral decompression seems logical in the treatment of proctalgia. For unilateral pain, the dilemma is more important. The risk is to induce pain at the "normal" side. Until now we have been performing bilateral operations without such a side effect but a controlled randomised study would be necessary to conclude.

The treatment of pain starts with a holistic approach of the woman (drugs, psychotherapy, relaxation...) with exclusion of other causes of pain: piriformis syndrome, coccygodynia, interstitial cystitis, endometriosis... The other neurological causes must be excluded by a complete electrophysiological study of the perineum (sacral latencies, PNTML, detection EMG and sensory evoked potentials) and imaging of the spinal cord [31]. If the diagnosis is confirmed an infiltration of the Alcock's canal under scanner control can be tried. This infiltration is successful in 57% in the short term but only in 15 % of the cases after one year [32]. It can be repeated maximum 3 times to avoid a nerve irritation. In the treatment of pain the results of this study are similar or better than those obtained in previous studies [32-34].

Even with the transgluteal approach where the "clamp" between the sacro-spinal and the sacro-tuberous ligament is opened by sectioning the sacro-spinal ligament, the cure rate remains around 50%. In the 4 cases of proctalgia fugax the results were better (3 cured and 1 improved). Shafik [5] had also very good outcomes with this type of pain (100% cured). In this study, the results were worse if the pain was bilateral.

For Robert early diagnosis appears to be the determining factor in improving results [35]. He used the infiltration of the Alcock's canal with lidocaïne-corticoïds as a test before operating. According to him a sufficient pain relieve, lasting during a short period, is a good indication for surgery. Mauillon et al also thinks that complete disappearance of pain for at least two weeks after a nerve block repeated twice before surgery may be the best criterion to predict success [34]. In this study, the patients presenting with perineodynia only (n = 10; Figure 1) had an infiltration before surgery but the number of cases was not sufficient to give a relevant impression about the infiltration test.

For anal incontinence, our results were in the same range as Shafik [29]. In a previous study we also had similar results [36,37]. The exclusion of the patients who had sphincteroplasty, levatorplasty (possible post-anal repair effect [27,28]) and/or a cure of rectocele did not change the cure rate.

More interesting was the cure rate in the group of patients with a clear rupture of one or the two anal sphincters. The traumatic rupture of the anal sphincter (delivery, sphincterotomy...) usually induces an immediate anal incontinence. In the patients who remained continent the power of the broken muscles remain sufficient to avoid flatus or faeces leakage. In the long term the continence is probably maintained with the help of the fibrous tissue located between the two edges of the ruptured muscle which acts like a bridge and therefore enables the sphincter to be efficient during many years. The aging process of the muscle and the pudendal neuropathy reduce the power of the muscle (and probably the sensibility in the anal canal) with time and explain the appearance of an anal incontinence. Therefore it is logical to restore continence by improving the conduction in the pudendal nerve. This fact can also explain why the results of sphincteroplasty decrease with time especially in the non diagnosed or treated pudendal neuropathies [38].

The fact that 5 patients were cured from their anal incontinence only 2 years after surgery emphasized the importance of a long follow up period to obtain relevant cure rates. Surprisingly, the cure rate seems to be not dependant of the degree of anal incontinence but the number of solid incontinences (5 cases; 3 cured and 2 improved) was too small to validate this impression.

The results of the pudendal nerve decompression seem to be equivalent to these of neuromodulation [39] and the procedure is far less expensive because there is no need for a special material. If this study is confirmed by others, the treatment of the neuropathy should be done before any trial of neuromodulation. In fact it is logical to repair the electric cable before enabling the current to pass.

For urinary incontinence the number of cases is too small to give a relevant cure rate but there were enough cases to suggest that this surgery can treat some patients with stress or urge incontinence.

In a previous study, 3 of the 7 patients presenting a stress urinary incontinence were cured by bilateral pudendal nerve decompression alone [36,37].

In Shafik's study 6 patients were cured from their stress urinary incontinence, 5 improved and one was a failure [30]. For this author, the efficacy of the pudendal nerve decompression on stress urinary incontinence is due to an increase of the external urethral sphincter EMG activity and to a decrease in the straining urethral reflex latency (time between the expiration involved with the cough and the first deflection of the reflex muscle action potential complex) and PNTML. For Shafik [40] the increase of urethral pressure during abdominal hyperpressure is not only passive but is induced by an active contraction of the urethral sphincter. After an injection of lidocaïne in the sphincter the urethral hyperpressure was suppressed. Thind & al. clearly demonstrated the role of the pudendal nerve in urinary continence. These authors showed a clear reduction of the maximum urethral pressure and a decrease in the adjunctive urethral closure forces during stress after bilateral pudendal blockade [41,42]. This is also in agreement with the study of Constantinou which demonstrated that a fast-acting contraction occurs in the distal third of the urethra 240 plus or minus 30 msec before the bladder hyperpressure [43]. Furthermore, Ko and Kim demonstrated that pudendal nerve block with a 7% phenol solution is very effective in the treatment of external urethral sphincter hypertonicity in patients with spinal cord injury [44].

This study is the first one dealing with a possible effect of the pudendal nerve decompression on urge incontinence. It is probably due to a better control of the urethral sphincter which can reduce urethral instability [45] and improve the inhibition of the detrusor activity.

One weakness of this study is the rough evaluation of the symptoms. We did not use any scoring system, pad test, quality of life questionnaires or "visual analog pain scale". Furthermore the number of anal and urethro-vesical manometries done before and after PND was too small to give relevant results.

The objective evaluation of PND was done using two neurophysiological tests and the clinical examination. Like Shafik [29,30] we found a significant increase in anal richness on EMG, a significant reduction of anal and perineal PNTML after surgery and a significant reduction in the frequency of the clinical signs. The skin rolling test was improved as much as the perineal sensibility and the Alcock's canal pain, thus showing its relevant link with the PCS.

### Evaluation of the clinical signs and minimal criteria needed for the diagnosis

Shafik described many clinical signs of the PCS [5,29,30]. In this study only three signs were studied carefully. Shafik described two of them: abnormal perineal sensibility and pain during the palpation of the Alcock's canal by a rectal examination. The third one is the "skin rolling test" which is well known in the diagnosis of neuralgia in other parts of the body [22]. This study is the first one in which this test was utilized as a clinical sign of PCS.

Compared to patients with negative clinical signs, those having positive clinical signs have a 4,42 ; 5,52 and 6,56 higher likelihood of PCS for "Abnormal sensibility", "Painful Alcock's canal" and "Painful skin rolling test", respectively (Table 10). When patients with all three signs positive are compared to patients with all three signs negative, the odd ratio is 16,97 (Table 11). All the estimated 95% confidence intervals for the odds ratios are significantly higher than 1, indicating that the clinical signs can be considered as valuable signs in the diagnosis of PCS.

The most specific sign was the "Painful skin rolling test" and the most sensitive was the "Painful Alcock's canal". The association of the three positive tests had a very high specificity in the diagnosis of a PCS (89 %). This high specificity was confirmed by the low frequency of this association after the operation (return to the same level as the control group).

Therefore, in some cases the clinical examination should be sufficient to prove the existence of a PCS. For example, a patient presenting anal incontinence, an intact sphincter proven by ultrasound and the three clinical signs positive has almost certainly a PCS. Of course, from a scientific point of view it is still interesting to perform a complete electrophysiological study and a precise neurological examination to exclude a central problem (multiple sclerosis, tumor...) or a polyneuropathy.

However, making the diagnosis of PCS is not usually an easy task. Many times, there is a high degree of suspicion but, as in many illness, all the symptoms or signs are not present. In this study, we decided to operate when at least two of the five clinical and neurophysiological signs described in the methods section were associated with one or more of the 3 classical symptoms (perineodynia, anal incontinence and urinary incontinence). At the beginning of this study, it was usually "increased PNTML" and "painful Alcock's canal". With the introduction of the "skin rolling test" and of the "sensibility test", clinical examination became more important in the decision. The more symptoms (especially anal incontinence and perineodynia) and signs were present, the more confident we were in the diagnosis of PCS. Further studies are necessary to validate this and to define more precisely the minimal criteria needed for the PCS diagnosis.

### Side effects

The pudendal nerve decompression by the perineal route is a blind procedure. The search for the inferior rectal nerve and the opening of the Alcock's canal are done under finger control. In our experience it is not easier with retractors. Therefore it is necessary to have a clear anatomical vision of this area before performing the operation.

Maybe the use of a laparoscope would help [46] but the procedure will become more expensive and time consuming. To suppress the blind character of the procedure the transgluteal approach proposed by Robert [8] or the more recent transvaginal approach from Bautrant [47] could be other ways to treat the PCS. Until now the results on pain are the same as those obtained by the Shafik's approach but with the concurrent sections of one or two ligaments of the pelvis (sacro-spinal and/or sacro-tuberous ligaments). However, we should be aware that the long term effects of these sections on the stability of the pelvic region are until yet unknown. Therefore, if the "clamp" must be open efforts should be done to open it without cutting a ligament. Up to now no data are available about a potential effect of the transgluteal or transvaginal procedures on urinary or anal incontinence.

Despite the blind character of the procedure we only had one heavy bleeding probably coming from the pudendal artery. One patient still presents with a mild intermittent clitoridal pain and a worsening of anal incontinence. Because the nerve of the clitoris leaves the pudendal nerve just before the entrance into the Alcock's canal this problem is probably the result of a too large dissection in the upper part of this canal. The two cases of anal incontinence worsening (gas incontinence becoming liquid incontinence), including the aforementioned patient, are difficult to explain. Maybe the neuropathy increased with the stretching involved in the procedure, the scarring process or a too large dissection. It could also be the result of the changes in the posterior level anatomy induced by concomitant procedures (easier expulsion of gas or faeces). For the 2 patients the EMG data and the clinical examination after the operation did not improve therefore showing that the neuropathy was not healing.

### Prevalence

Because the roughly estimate prevalence of PCS is around 20%, this "defect" seems to be a very frequent problem in Perineology. Therefore it should be logical to search for it in each clinical examination of a patient presenting with prolapse, perineodynia, urinary or anal incontinence.

## Conclusions

Pudendal neuropathy is probably a frequent "defect" in perineology. Pudendal nerve decompression seems to be the defect specific procedure indicated in such a problem. In fact it can treat perineodynia, anal and probably urinary incontinence. Anal incontinence can be cured by pudendal nerve decompression alone even in the presence of a clear disruption of the anal sphincter on anal ultrasound. Anal richness on EMG increases and PNTML decrease significantly after surgery proving an objective effect on the nerve. The frequency of abnormal puncture sensibility, painful Alcock's canal and painful "skin rolling test" are significantly reduced by the operation. This study suggests that the three clinical tests could be used in practice to confirm or suspect the diagnosis of pudendal neuropathy in case of pain, urinary and/or anal incontinence. However, further studies are necessary to confirm these preliminary results.

## Abbreviations

PCS = pudendal canal syndrome

PND = pudendal nerve decompression

EMG = electromyography

PNTML = pudendal nerve terminal motor latency

## Competing interests

The authors declare that they have no competing interests.

## Authors' contribution

JB conceived the study, carried out surgery and clinical follow up and drafted the manuscript.

DC participated in the design of the study and performed the statistical analysis.

MB carried out the neurophysiological examinations.

All authors read and approved the final manuscript.

## Pre-publication history

The pre-publication history for this paper can be accessed here:


